# Putative functional non-coding polymorphisms in *SELP* significantly modulate sP-selectin levels, arterial stiffness and type 2 diabetes mellitus susceptibility

**DOI:** 10.1186/s12902-020-00548-x

**Published:** 2020-05-19

**Authors:** Raminderjit Kaur, Jatinder Singh, Rohit Kapoor, Manpreet Kaur

**Affiliations:** 1 School of Medicine, Wayne State University, Detroit, Michigan USA; 2 Department of Molecular Biology & Biochemistry, Guru Nanak Dev University, Amritsar, Punjab India; 3 Carewell Heart & Superspeciality Hospital, Amritsar, Punjab India

**Keywords:** Atherosclerosis, Haplotype, Pulse wave velocity, Selectin, SNP, Vascular risk

## Abstract

**Background:**

P-selectin, encoded by *SELP*, has been implicated as an important molecule in the development of arterial stiffness, consequently leading to vascular complications in T2DM. *SELP* polymorphisms and increased levels of soluble P-selectin (sP-selectin) have been shown to be associated with several inflammatory diseases. The present work was designed to assess nine putative functional non-coding *SELP* variants in relation to sP-selectin levels and arterial stiffness in T2DM.

**Methods:**

The genetic distribution of rs3917655, rs3917657, rs3917739, rs2235302, rs3917843 was determined by restriction fragment length polymorphism–polymerase chain reaction (RFLP-PCR). Genotyping of rs3917779 was performed by tetra primer amplification-refractory mutation system (ARMS)- PCR. Three SNPs i.e. rs3917853, rs3917854, rs3917855 were genotyped by Sanger sequencing. Construction of haplotypes was performed using PHASE software. The data thus obtained was analyzed by appropriate statistical tools.

**Results:**

Two non-coding variants i.e. rs3917657 and rs3917854 of *SELP* were found to be associated with 2 and 1.7 -fold risk of disease development respectively. However, one non-coding variant rs2235302 was found to provide protection against disease development. Furthermore, variant allele of rs3917854 in T2DM patients was found to be associated with 2.07-fold very high vascular risk. Non-coding haplotype GCAGGCCGC was conferring 4.14-fold risk of disease development. Furthermore, overall sP-selectin levels were higher in T2DM patients when segregated according to genotypes as well as haplotypes. Significant genotype- phenotype correlation was observed for rs3917655 as well as rs3917739 variant in patients and for rs3917854 in controls. In vascular risk categories, a significant genotype- phenotype correlation was observed for rs3917655 and rs2235302. Furthermore, patients with CCGGGCCGC haplotype in high risk category were observed with higher levels of sP-selectin as compared to other haplotypes (*p* < 0.05).

**Conclusions:**

Non-coding *SELP* variants may significantly modulate sP-selectin levels, vascular risk and T2DM susceptibility.

## Background

Atherosclerosis is the major contributing factor for vascular complications, leading to high rate of mortality and morbidity in T2DM [1, 2]. Atherosclerosis causes degeneration of arterial elasticity, resulting in arterial stiffness, which is a key risk factor for the development of nephropathy, myocardial infarction (MI), stroke and other vascular complications in T2DM patients [3–7]. In addition, advanced glycation end products (AGE) are also generated in an accelerated manner in diabetes as well as in pre-diabetes conditions [8, 9]. AGE-RAGE (receptor of AGE) axis has been shown to modulate inflammatory cascade, contributing to cardiovascular damage in these conditions [10].

Pulse wave velocity (PWV), a non-invasive method, is widely used for the assessment of arterial stiffness [11]. Brachial-ankle PWV (baPWV) has been extensively used for the detection of augmented arterial stiffness in a large population and is suggested as an independent predictor of atherosclerotic vascular damage and cardiovascular risk [12–17]. Arterial stiffness is considered to be a low-grade inflammatory condition [18, 19]. Inflammatory response is characterized by translocation of the adhesion molecules, such as selectins to the surface, initiating the adhesion cascade for leukocyte recruitment to the vascular wall [20]. P-selectin, largest among the other selectins, is a key mediator of leukocyte, platelet and endothelium interactions. Binding of P-selectin to its ligands mediate initial steps of adhesion cascade i.e. tethering and rolling [21, 22]. This interaction further results into proteolytic shedding of P-selectin in circulation as soluble P-selectin (sP-selectin), which is documented as marker of endothelial dysfunction and platelet hyperactivity [23–27]. Furthermore, studies have suggested a significant association of raised sP-selectin levels with atherosclerotic vascular complications including coronary heart disease (CHD), CAD and MI in T2DM [26, 28–32].

*SELP*, a gene encoding P-selectin, variations have been suggested to contribute towards susceptibility to arterial stiffness and vascular complications. Furthermore, inactivation of *SELP* in atherosclerosis prone mouse models led to decreased formation of atherosclerotic plaques [33]. Several single-nucleotide polymorphisms (SNPs) of *SELP* have been shown to be associated with risk of different atherosclerotic as well as inflammatory diseases, including diabetic retinopathy, T2DM, CAD, CHD, ischemic stroke and systemic lupus erythematous, peripheral artery disease in different populations [26, 34–42]. Furthermore, *SELP* variants were also reported to be associated with modulations in sP-selectin levels in different atherosclerotic vascular complications [26, 36, 43–48]. Most of the available reports have evaluated the clinical relevance of only coding region variants of *SELP.* The non-coding variants can also have detrimental effect on phenotypic expression of a gene. Only three non-coding SNPs of *SELP* i.e. rs3917657, rs2235302 and rs3917779, were previously found to be associated with systemic lupus erythematosus (SLE), carotid intima-media thickness and diabetic retinopathy [48–51]. These variants may alter the gene expression by affecting transcription factor binding sites, splicing regulation and miRNA binding etc. [52].

Due to population-specific nature of association studies, there is a universal need to replicate the studies in different populations. So, the present study was designed to investigate role of non-coding SNPs as important genetic markers in T2DM. All the selected variants were documented to have putative functional role in our previous study [53]. As per literature survey, this is the first comprehensive study evaluating nine putative functional non-coding *SELP* variants in relation to sP-selectin levels, arterial stiffness and T2DM susceptibility.

## Methods

### Study participants

A total of 250 T2DM patients comprising 99 females and 152 males, with HbA1c ≥6.5%, aged 30–80 y and from Carewell Heart & Superspeciality Hospital, Amritsar (PB), were enrolled for the present case-control study. HbA1c levels of patients were determined using fully automated Alere Afinion™ analyzer by manufacturer’s protocol (Afinion-AS100, Alera Technologies AS, Norway). Gender- and Age- matched 264 healthy controls (having fasting glucose < 100 mg/dl or HbA1c < 5.7%) including 107 females and 157 males were also recruited from the adjoining areas. The details regarding demographic characteristics, disease history and arterial stiffness assessment as well as vascular risk stratification in T2DM patients has already been explained previously [26, 54]. The blood samples were collected and processed for DNA and serum isolation [26].

### Genotyping of SELP variants

A total of nine SNPs selected on the basis of in silico analyses were genotyped by various methods including RFLP-PCR, ARMS-PCR and Sanger sequencing. Genotyping of five variants i.e. rs3917655, rs3917657, rs3917739, rs3917843 and rs2235302, was performed using PCR-RFLP. Components and conditions used in PCR-RFLP of these SNPs are specified in Table 1. The details of various components used for restriction digestion reaction of the abovesaid variants are specified in supplementary table 1. Genotyping of rs3917779 was carried out using tetra primer ARMS-PCR. The primers used for tetra primer ARMS-PCR were T allele specific forward inner primer (GAATCTCAGGTAAGTCACTTGTGAATTGAT); reverse outer primer (TTTCCTAATGGCACATGACTTGGAG); C allele specific reverse inner primer (GCTGCAATCTGTGGAGTGGAAAATAG) and forward outer primer (TCCACACAAATGACCCTTAAGTTGG). The PCR conditions, including denaturation at 94 °C for 7 min, followed by 35 cycles each of 30 s at 94 °C for denaturation, at 63 °C for annealing, at 72 °C for extension and, a final extension step at 72 °C for 7 min. The PCR products with expected size 441 bp, 254 bp (T allele) and 243 bp (C allele) were examined on 1.5% (w/v) agarose gel pre-stained with ethidium bromide (EtBr). The details of PCR components are specified in supplementary table 2. The remaining three non-coding SNPs i.e.*,* rs3917853, rs3917854, rs3917855 were genotyped using Sanger sequencing (*n* = 233). Due to paucity of funds, we were unable to perform sequencing of complete 514 subjects. The primers used for Sanger sequencing were forward primer (5’GCATTTGACCCGAGTCCTTA3’) and reverse primer (5’AGGAAAAGGACAGGTCTCTGGA3’). The PCR conditions, including denaturation at 94 °C for 7 min, followed by 35 cycles each of 30 s at 94 °C for denaturation, at 64 °C for annealing, at 72 °C for extension and, a final extension step at 72 °C for 7 min. The PCR products with expected size 620 bp were determined on 1.5% (w/v) agarose gel pre-stained with EtBr. 10% of indicative samples of each SNP having various genotypes i.e.*,* wild, variant and heterozygous were subjected to Sanger sequencing and concordance rate between genotyping by PCR-RFLP and sanger sequencing was 100%.

**Table 1:** Components and conditions used in PCR-RFLP of rs3917655, rs3917657, rs3917739, rs3917843 and rs223530

SNP	Primer sequence	PCR conditions	Amplicone Size (bp)	RFLP			
				Restriction enzymes	Incubation conditions	Product after digestion (bp)	
						Ancestral	Variant
rs3917655	5’TGTCCACTTTGACCCTCCCA3’ 5’AGGGCAGAAAAGGAAACTATGTG3’	Initial denaturation at 95 °C (7 min) 30 s at 95 °C 30 s at 58 °C 30 s at 72 °C Final elongation at 72 °C for 7 min	405	*PvuII*	At 37 °C for 2 h	249	405
						156	
rs3917657	5’ATCTTCTGGGACTGATCTGGA3’ 5’CCTGCCTGGTTCCTCCATAG3’	Initial denaturation at 95 °C (7 min) 30 s at 95 °C 30 s at 60 °C 30 s at 72 °C Final elongation at 72 °C for 7 min	516	*TfiI*	At 65 °C for 2 h	265	265
						251	199 52
rs3917739	5’AAAGCCCAGAGCAAAGAGGTAGT3’ 5’CCCTCCCTTCCCACCTTAACT3’	Initial denaturation at 95 °C (7 min) 30 s at 95 °C 30 s at 60 °C 30 s at 72 °C Final elongation at 72 °C for 7 min	546	*TfiI*	At 65 °C for 2 h	546	328
							218
rs3917843	5’ATTACATGCAATGCCTGCCT3’ 5’GGGGCATACTGTCCCTTTTTGA3’	Initial denaturation at 95 °C (7 min) 30 s at 95 °C 30 s at 59 °C 30 s at 72 °C Final elongation at 72 °C for 7 min	578	*BsaWI*	At 60 °C for 2 h	329	578
						249	
rs2235302	5’GCCAACCTGTGAGGGTAGGAT3’ 5’ACCACTGTCCGCCTTATAAACT3’	Initial denaturation at 95 °C (7 min) 30 s at 95 °C 30 s at 57 °C 30 s at 72 °C Final elongation at 72 °C for 7 min	511	*EciI*	At 37 °C for 2 h	441	511
						70	

PCR and digestion products were analyzed on 1.5 and 2.5% agarose gel pertained with EtBr, respectively

### Evaluation of sP-selectin levels

Serum sP-selectin levels were measured by ELISA, according to manufacturer’s instructions (RayBiotech, USA) as discussed previously [26].

### Statistical analyses

Sample size calculation was for genetic association was calculated using CaTS power calculator (http://csg.sph.umich.edu/abecasis/CaTS/) as explained in our previous report [26, 55]. Comparison of genotypic and allelic frequencies between groups was carried out by Odds ratio using MedCalc software (https://www.medcalc.org/). Genetic models were determined by Web-Asso test program (http://www.asso-web.com/). Construction of haplotypes was carried out by PHASE software version 2.1.1 [56]. Linkage disequilibrium (LD) was determined by Haploview version 4.2 [57]. One-way ANOVA followed by Tukey’s multiple comparison post hoc-test were used to compare sP-selectin levels (mean ± SD). Student’s t-test was used to compare sP-selectin levels in different genotypic or haplotype combinations between the studied groups. Whole data was analyzed to remove the outliers using Box whisker plot. Various statistical analyses were carried out using SPSS version 16.0 (IL, USA and Chicago). For the whole analyses, *p* value < 0.05 was taken as statistically significant.

## Results

Out of nine non-coding variants, two variants i.e. rs3917657 and rs3917854 were found to be associated with risk, while one variant rs2235302 showed protection towards disease development. The representative agarose gels showing PCR products and restriction digestion products as well as electropherograms of representative samples for all the studied variants are given in supplementary figure 1–**7**. Due to low frequency (*n* ≤ 2) of homozygous variant and heterozygous genotypes of rs3917853 and rs3917855, these were excluded from further statistical analyses. Genotypic and allelic distribution was significant different for rs3917657 between patients and controls (Table 2). Heterozygosity and variant allele frequency were significantly more prevalent in patients with 1.9 -fold risk of T2DM. After adjustment for confounding factors of T2DM, the risk was marginally increased (Table 2). The association was indicated in dominant (CT/TT vs.CC; OR-1.98, 95% CI-1.26-3.11, *p* = 0.003) as well as co-dominant (TT/CT = CT/CC; OR-1.88, 95% CI-1.24-2.85, *p* = 0.002) models. For rs3917854, significantly high frequency of homozygous variant genotype was observed in patients, representing 2.4-fold risk of disease development (Table 2), which was marginally increased after confounding factors adjustment (Table 2). The variant allele was found to confer 1.7-fold risk of disease development. The association was indicated in co-dominant model (AA/GA = GA/GG; OR-1.64, 95% CI-1.12-2.41, *P* − 0.009). Genotypic and allelic distribution of rs2235302 was observed to be significantly different between patients and controls (Table 2). The frequency of homozygous variant genotype was significantly low in patients as compared to controls and was associated with protection. Marginally increased effect was observed after adjustment for confounding variables (Table 2). Similar heterozygosity distribution was obtained in both studied groups. The variant allele showed the protective association with disease development. There were suggestive evidences of an association of T2DM with co-dominant model (AA/GA = GA/GG; OR-0.75, 95% CI-0.57-0.97, *p* = 0.034). High frequency of homozygous variant genotype as well as variant allele was observed for rs3917655 and rs3917739. However, the differences were not statistically significant. Similar genotypic as well as allelic frequency distribution was observed for rs3917843. In case of rs3917779, high prevalence of homozygous wild genotype was observed in both patients and controls. However, homozygous variant genotype was completely absent in both the studied groups.

**Table 2:** Genetic distribution of non-coding variants in patients and controls along with genetic models

Variants	Patients N (%)	Controls N (%)	Crude		Adjusted		Dominant Model OR (95% CI) ***p*** value	Co-dominant Model OR (95% CI) ***p*** value	Recessive Model O R (95% CI) ***p*** value
			OR (95% CI)	***p*** value	OR	***p*** value			
**rs3917655 Genotypes**									
**CC**	132 (52.8)	149 (56.44)	reference				1.16 (0.82 to1.64) 0.407	1.19 (0.89 to 1.59) 0.235	1.68 (0.77 to 3.66) 0.187
**CG**	101 (40.4)	104 (39.39)	1.10 (0.76 to 1.57)	0.620	0.95	0.827			
**GG**	17 (6.8)	11 (4.17)	1.74 (0.79 to 3.86)	0.170	1.70	0.259			
**Alleles**									
**C**	365 (73)	402 (76.14)	reference						
**G**	135 (27)	126 (23.86)	1.18 (0.89 to 1.56)	0.250					
**rs3917657 Genotypes**									
**CC**	189 (75.6)	227 (85.98)	reference				1.98 (1.26 to 3.11) 0.003**	1.88 (1.24 to 2.85) 0.002**	2.67 (0.51 to 13.91) 0.218
**CT**	56 (22.4)	35 (13.25)	1.92 (1.20 to 3.05)	0.005**	1.94	0.014*			
**TT**	5 (2)	2 (0.7)	3.00 (0.58 to 15.65)	0.191	3.16	0.214			
**Alleles**									
**C**	434 (86.8)	489 (92.61)	reference						
**T**	66 (26.4)	39 (14.77)	1.91(1.26 to 2.89)	0.002**					
**rs3917739 Genotypes**									
**GG**	31 (12.4)	39 (14.77)	reference				1.22 (0.74 to 2.03) 0.433	1.21 (0.94 to 1.56) 0.311	1.31 (0.92 to 1.87) 0.135
**GA**	111(44.4)	128 (48.48)	1.09 (0.64 to 1.86)	0.750	1.24	0.483			
**AA**	108 (43.2)	97 (36.74)	1.4 (0.81 to 2.41)	0.230	1.51	0.188			
**Alleles**									
**G**	173 (34.6)	206 (39.01)	reference						
**A**	327 (65.4)	322 (60.98)	1.21 (0.94 to 1.56)	0.140					
**rs3917843 Genotypes**									
**GG**	183 (73.2)	186 (70.45)	reference				0.87 (0.59 to 1.28) 0.489	0.89 (0.63 to 1.25) 0.497	0.88 (0.26 to 2.91) 0.831
**GA**	62 (24.8)	72 (27.27)	0.87 (0.59 to 1.3)	0.511	1.24	0.483			
**AA**	5 (2)	6 (2.27)	0.85 (0.25 to 2.82)	0.792	1.51	0.188			
**Alleles**									
**G**	428 (81.06)	444 (84.09)	reference						
**A**	72 (13.64)	84 (15.90)	0.89 (0.63 to 1.25)	0.500					
**rs2235302 Genotypes**									
**GG**	98 (39.2)	86 (32.57)	reference				0.75 (0.52 to 1.08) 0.125	0.75 (0.57 to 0.98) 0.034*	0.58 (0.34 to 1.01) 0.049*
**GA**	129 (51.6)	138 (52.27)	0.82 (0.56 to 1.19)	0.300	0.79	0.284			
**AA**	23 (9.2)	39 (14.77)	0.50 (0.28 to 0.91)	0.023*	0.54	0.046*			
**Alleles**									
**G**	325 (65)	310 (58.71)	reference						
**A**	175 (35)	218 (41.29)	0.76 (0.59 to 0.98)	0.038*					
**rs3917779 Genotypes**									
**CC**	240 (96)	249 (94.32)	reference				–	–	–
**CT**	10 (4)	15 (5.68)	0.69 (0.30 to 1.57)	0.380	0.492	0.134			
**TT**	–	–	–						
**Alleles**									
**C**	490 (98)	513 (97.16)	reference						
**T**	10 (2)	15 (2.84)	0.70 (0.31 to 1.57)	0.384					
**rs3917854 Genotypes**									
**GG**	50 (42.73)	66 (56.89)	reference				1.77 (1.05 to 0.97) 0.030*	1.64 (1.12 to 2.41) 0.009**	2.45 (1.07 to 5.64) 0.027*
**GA**	47 (40.17)	41 (36.20)	1.51 (0.86 to 2.64)	0.140	1.32	0.386			
**AA**	20 (17.09)	9 (7.75)	2.93 (1.23 to 6.98)	0.015*	2.96	0.030*			
**Alleles**									
**G**	147 (62.82)	173 (74.56)	reference						
**A**	87 (37.17)	59 (25.43)	1.73 (1.16 to 2.58)	0.006**					

*OR* represents odds ratio, *CI* represents confidence interval; * represents *p* value significant at 0.05 level; ** represents *p* value significant at 0.01 level

To assess the effect of *SELP* variants on vascular risk, their frequency distribution was also compared between the vascular risk categories (Table 3). In variant rs3917657, rs3917843 and rs3917779, heterozygous variants and homozygous variants were combined to compute odds ratios as the frequency of homozygous variants is lesser i.e. < 5% in all the vascular risk categories. Out of all the variants, variant allele rs3917854 was found to be associated with 2-fold very high vascular risk, with significantly high frequency in very high risk (46.43%) than high risk category (29.55%). However, no significant difference in genotypic as well as allelic distribution was observed for other variants. Furthermore, these genotypic associations remained unaffected even after adjustment for various confounding factors of vascular risk (including age, gender, BMI, WHR, WSR, MAP, PP, LDL-C and VLDL) (data not shown).

**Table 3:** Comparison of genotypic/ allelic distribution of non-coding *SELP* variants between vascular risk categories

***SELP*** SNPs	Very high risk category N (%)	High risk category N (%)	Moderate risk category N (%)	Odds ratio (95% CI)			***p*** value		
				Very high risk vs. high risk	High risk vs. moderate risk	Very high risk vs. moderate risk	p^**a**^	p^**b**^	p^**c**^
**rs3917655 genotypes**									
**GG**	30 (55.55)	56 (51.37)	46 (53.48)	1	1	1			
**GA**	20 (37.03)	46 (42.20)	34 (39.53)	0.81 (0.40 to 1.61)	1.11 (0.61 to 2.00)	0.90 (0.44 to 1.85)	0.550	0.720	0.771
**AA**	4 (7.40)	7 (6.42)	6 (6.97)	1.07 (0.29 to 3.93)	0.96 (0.30 to 3.05)	1.02 (0.26 to 3.93)	0.920	0.940	0.970
**Alleles**									
**G**	80 (74.07)	158 (72.47)	126 (73.25)	1	1	1			
**A**	28 (25.93)	60 (27.53)	46 (26.75)	0.92 (0.54 to 1.55)	1.04 (0.66 to 1.63)	0.96 (0.55 to 1.65)	0.750	0.860	0.870
**rs3917657 genotypes**									
**CC**	38 (70.37)	81 (74.31)	69 (80.23)	1	1	1			
**CT**	15 (27.77)	26 (23.8)	15 (17.44)	1.23 (0.58 to 2.58)	1.47 (0.72 to 3.00)	1.81 (0.80 to 4.11)	0.581	0.282	0.150
CT + TT	16 (29.62)	28 (26.16)	17 (19.76)	1.22 (0.59 to 2.51)	1.40 (0.71 to 2.77)	1.71 (0.78 to 3.76)	0.593	0.331	0.183
**Alleles**									
**C**	91 (84.25)	188 (86.23)	153 (88.95)	1	1	1			
**T**	17 (15.75)	30 (13.77)	19 (11.05)	1.17 (0.61 to 2.23)	1.28 (0.69 to 2.37)	1.50 (0.74 to 3.04)	0.630	0.420	0.250
**rs3917739 genotypes**									
**GG**	6 (11.11)	11 (10.09)	14 (16.27)	1	1	1			
**GA**	28 (51.85)	52 (47.70)	31 (36.04)	0.99 (0.33 to 2.95)	2.13 (0.86 to 5.28)	2.11 (0.71 to 6.23)	0.980	0.101	0.171
**AA**	20 (37.03)	46 (42.20)	41 (47.67)	0.79 (0.26 to 2.45)	1.43 (0.58 to 3.49)	1.14 (0.38 to 3.40)	0.690	0.432	0.810
**Alleles**									
**G**	40 (37.03)	74 (33.94)	59 (34.30)	1	1	1			
**A**	68 (62.97)	144 (66.06)	113 (65.7)	0.87 (0.54 to 1.41)	1.02 (0.66 to 1.54)	0.89 (0.54 to 1.46)	0.581	0.940	0.643
**rs3917843 genotypes**									
**GG**	35 (64.81)	81 (74.31)	66 (76.74)	1	1	1			
**GA**	19 (35.18)	24 (22.01)	19 (22.09)	1.83 (0.89 to 3.76)	0.97 (0.49 to 1.92)	1.88 (0.88to 4.01)	0.099	0.934	0.100
GA + AA	19 (35.18)	28 (26.16)	20 (23.25)	1.57 (0.78 to 3.17)	1.14 (0.59 to 2.20)	1.79 (0.84 to 3.79)	0.209	0.695	0.127
**Alleles**									
**G**	89 (82.40)	186 (85.32)	151 (87.79)	1	1	1			
**A**	19 (17.6)	32 (14.68)	21 (12.21)	1.24 (0.66 to 2.31)	1.23 (0.68 to 2.23)	1.53 (0.78 to 3.01)	0.496	0.480	0.212
**rs2235302 genotypes**									
**GG**	21 (38.88)	42 (38.53)	35 (40.69)	1	1	1			
**GA**	31 (57.40)	55 (50.45)	42 (48.83)	1.13 (0.57 to 2.23)	1.09 (0.59 to 1.99)	1.23 (0.60 to 2.50)	0.730	0.770	0.561
**AA**	2 (3.70)	12 (11.00)	9 (10.46)	0.33 (0.07 to 1.63)	1.11 (0.42 to 2.94)	0.37 (0.07 to 1.88)	0.171	0.832	0.230
**Alleles**									
**G**	73 (67.59)	139 (63.76)	112 (65.11)	1	1	1			
**A**	35 (32.41)	79 (36.24)	60 (34.89)	0.84 (0.52 to 1.37)	1.06 (0.69 to 1.61)	0.89 (0.53 to 1.49)	0.490	0.783	0.673
**rs3917779 genotypes**									
**CC**	53 (98.24)	107 (98.15)	79 (91.86)	1	1	1			
CT	1 (1.76)	2 (1.85)	7 (8.14)	1.00 (0.08 to 11.38)	0.21 (0.04 to 1.04)	4.70 (0.56 to 39.28)	0.620	0.056	0.150
CT + TT	1 (1.76)	2 (1.85)	7 (8.14)	1.00 (0.08 to 11.38)	0.21 (0.04 to 1.04)	4.70 (0.56 to 39.28)	0.620	0.056	0.150
**Alleles**									
**C**	107 (99.07)	216 (99.08)	165 (95.93)	1	1	1			
**T**	1 (0.93)	2 (0.92)	7 (4.07)	2.01 (0.12 to 32.44)	0.21 (0.04 to 1.06)	4.54 (0.55 to 37.42)	0.622	0.059	0.160
**rs3917854 genotypes**									
**GG**	8 (28.57)	23 (52.27)	19 (42.22)	1	1	1			
**GA**	14 (50)	16 (36.36)	17 (37.77)	2.51 (0.86 to 7.39)	0.77 (0.312 to 1.94)	1.95 (0.66 to 5.80)	0.093	0.589	0.226
**AA**	6 (21.4)	5 (11.36)	9 (20)	3.45 (0.82 to 14.47)	0.46 (0.13 to 1.60)	1.58 (0.42 to 5.94)	0.090	0.222	0.495
**Alleles**									
**G**	30 (53.57)	62 (70.45)	55 (61.11)	1	1	1			
**A**	26 (46.43)	26 (29.55)	35 (38.89)	2.07 (1.03 to 4.15)	0.66 (0.35 to 1.23)	1.36 (0.69 to 2.67)	0.041^a^		0.369

^a^represents *p* value significant at 0.05 level pa denotes for *p* value of comparison between very high risk and high risk category; p^b^ denotes for *p* value of comparison between high risk and moderate risk category; p^c^ denotes for *p* value of comparison between very high risk and moderate risk category

**Fig. 1:**
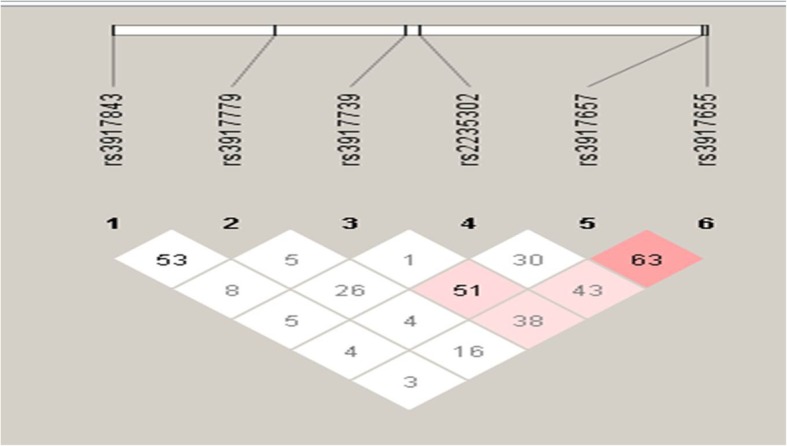
LD plot of *SELP* variants. The dark pink squares represents intermediate LD (D’ = 0.632; LOD > 2.00), light pink squares represents low LD (D’ = 0.511, 0.430 and 0.388; LOD > 2.00) and white/blue squares indicates non-significant LD (LOD < 2.00)

For all the studied SNPs, deviation from Hardy–Weinberg was tested using Web-asso test. All genotypes were distributed according to HWE in controls (all *p* values were more than 0.05). LD is generally determined by D’ value and LOD score. The D’ value is ranged from 0 to1, where 0 designates complete equilibrium and 1 specifies complete LD. LOD represents log of the odds of there being LD between two loci and LOD score ≥ 2.0 is normally considered as a significant evidence of LD. In the present study, three variants i.e. rs3917853, rs3917854, rs3917855 were excluded form LD analysis due to low statistical power. One SNP pair i.e. rs3917655/rs3917657 was observed with intermediate LD with D’/ LOD values 0.632/15.71 **(**Fig. 1). Three SNP pairs i.e. rs3917739/rs3917657, rs3917655/rs2235302 and rs3917655/rs3917739 were observed to have low LD with D’/ LOD values 0.511/2.6, 0.430/9.81 and 0.388/3.33 respectively.

Haplotypes of *SELP* variants were constructed and their frequencies were compared in both the studied groups. The order of SNPs in the haplotypes was as follows: rs3917655, rs3917657, rs3917739, rs3917843, rs2235302, rs3917779, rs3917853, rs3917854, rs3917855. Out of 29 haplotypes, only 18 haplotypes with frequency ≥ 0.01 in any of the studied group were subjected to further statistical analyses (Table 4**)**. Being most prevalent in both the studied groups, CCAGGCCGC haplotype was taken as reference for further analysis. Three haplotypes i.e. CCAGGCCAC, GCAGGCCGC, GTAGACCGC were observed at higher frequencies (> 0.05) in patients than controls. Out of these, only GCAGGCCGC haplotype was observed to be associated with 4-fold risk. Although not statistically significant, CCGGGCCGC, CCGGGCCAC, CCGGACCGC, GCAGACCGC haplotypes were less prevalent in patients (*p* = 0.05).

**Table 4:** Comparison of non-coding haplotype distribution between patients and controls

Haplotypes	Patients (N)	Freq. (2 ***N*** = 234)	Controls (N)	Freq. (2 ***N*** = 232)	OR	95% CI	***p*** value
CCAGGCCGC	38	0.1623	45	0.1939	1	1	
CCAGGCCAC	31	0.1324	21	0.0905	1.74	0.86 to 3.53	0.119
CCGGGCCGC	20	0.0854	39	0.1681	0. 61	0.30 to 1.21	0.157
CCGGGCCAC	18	0.0769	19	0.0818	1.13	0.52 to 2.43	0.771
GCAGGCCGC	14	0.0598	4	0.0172	4.14	1.25 to 13.65	0.019^a^
GTAGACCGC	14	0.0598	6	0.0258	2.76	0.96 to 7.89	0.057
CCGGACCGC	11	0.0470	15	0.0646	0.86	0.35 to 2.11	0.756
CCGGACCAC	9	0.0384	8	0.0344	1.33	0.46 to 3.79	0.590
CCAGACCGC	9	0.0384	5	0.0215	2.13	0.65 to 6.90	0.206
CTAGGCCGC	7	0.0299	2	0.0086	4.14	0.81 to 21.14	0.087
GCAGACCGC	7	0.0299	19	0.0818	0.43	0.16 to 1.14	0.093
CCGAGCCGC	6	0.0256	4	0.0172	1.77	0.46 to 6.76	0.399
GCAAACCGC	6	0.0256	8	0.0344	0.88	0.28 to 2.78	0.838
CCGAGCCAC	4	0.0170	2	0.0086	2.36	0.41 to 13.64	0.334
CCAAACCGC	3	0.0128	2	0.0086	1.77	0.28 to 11.19	0.540
GCGGACCGC	3	0.0128	3	0.0129	1.18	0.22 to 6.21	0.841
CCAAGCCGC	2	0.0085	4	0.0172	0.59	0.10 to 3.41	0.557
GCAGGTCGC	1	0.0042	3	0.0129	0.39	0.03 to 3.95	0.429

*OR* denotes for odds ratio, *CI* denotes for confidence interval; ^a^represents statistical significance at 0.05 level, *Freq.* denotes for frequency, *N* denotes for number

When segregated into vascular risk categories, nine haplotypes were observed with frequencies ≥0.01 in any of the risk category. As CCAGGCCGC was the most prevalent (> 0.1) haplotype in two of the three categories, it was selected as the reference haplotype (data not shown). However, no statistically significant difference was found in vascular risk categories (*p* > 0.05). The other prevalent haplotypes in these risk categories were CCAGGCCAC (16%; 12.5%; 12.2%), followed by CCGGGCCGC (14.3%; 11.4%; 7.7%) and CCGGGCCAC (10.7%; 5.6%; 10%).

**Fig. 2:**
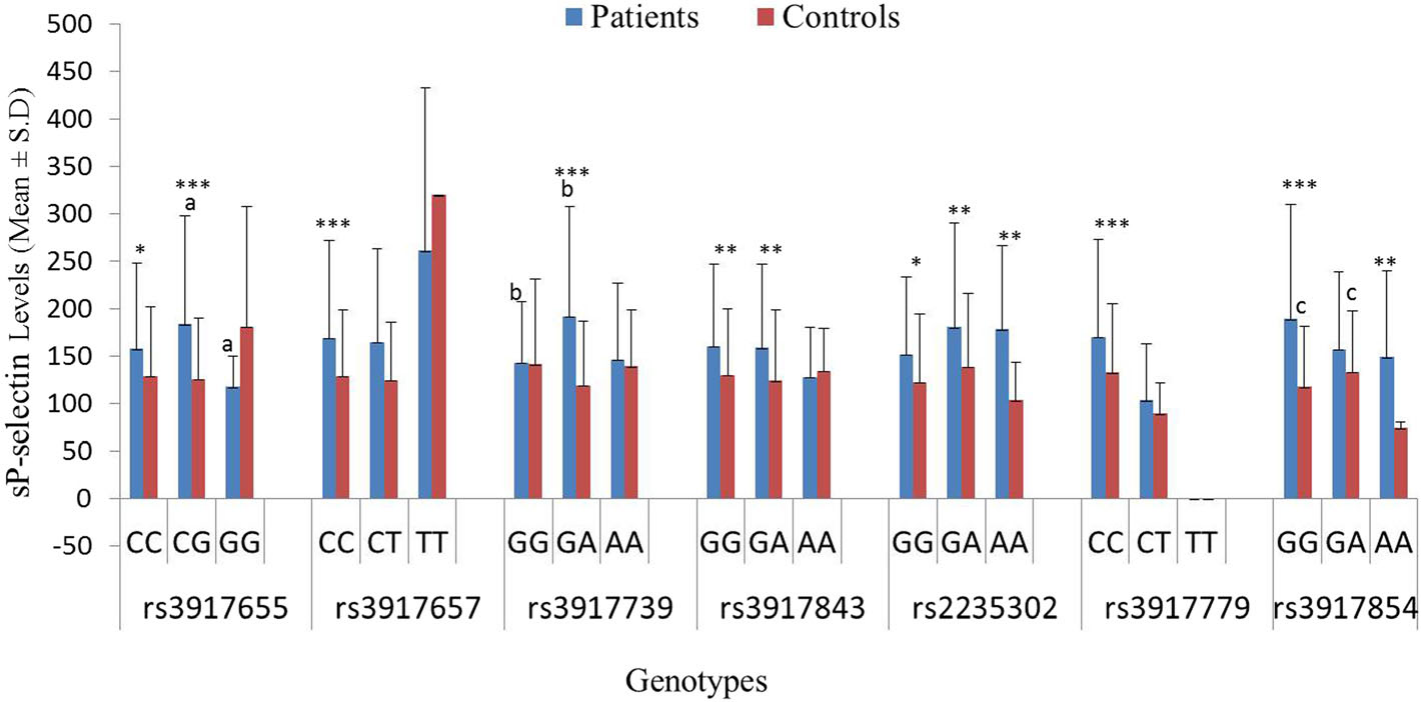
Comparisons of sP-selectin levels between T2DM patients and controls stratified according to genotypes. Lowercase letters represent comparison within the groups; ^a^*p* = 0.047, ^b^*p* = 0.005; ^c^*p* = 0.027; * represents significance at 0.05 level between the groups; ** represents significance at 0.01 level between the groups, ***represents significance at 0.001 level between the groups

In our previous study, patients showed significantly high sP-selectin levels as compared to controls (*p* < 0.001) [26]. For rs3917655, patients with heterozygous genotype were observed with significantly high sP-selectin levels than patients with homozygous variant genotype (*p* < 0.05) (Fig. 2). Furthermore, patients with homozygous wild and heterozygous genotypes had significantly high sP-selectin levels (*p* < 0.05; < 0.001 respectively) than controls with the respective genotypes. Only homozygous wild genotype accounted for significantly raised levels of sP-selectin (*p* < 0.001) in patients as compared to controls for rs3917657. Furthermore, in rs3917739, a significant difference was observed in sP-selectin levels only within the patients, where heterozygous genotype was accounted for significantly high levels as compared to homozygous wild genotype (*p* < 0.01). Similar results were observed when heterozygous genotype of patients was compared with respective genotype of controls (*p* < 0.001). Furthermore, in case of rs3917843 and rs2235302, no significant difference was observed within the studied groups (*p* < 0.05). Patients with homozygous wild as well as heterozygous genotypes of rs3917843, all genotypes of rs2235302 and homozygous wild genotype of rs3917779 were found to have significantly high sP-selectin levels as compared to respective controls. For rs3917854, significantly high sP-selectin levels were observed in controls with heterozygous genotype than homozygous wild genotype. Patients with homozygous wild as well as variant genotypes were observed to have significantly high sP-selectin levels as compared to respective controls (*p* < 0.001; < 0.01 respectively).

**Fig. 3:**
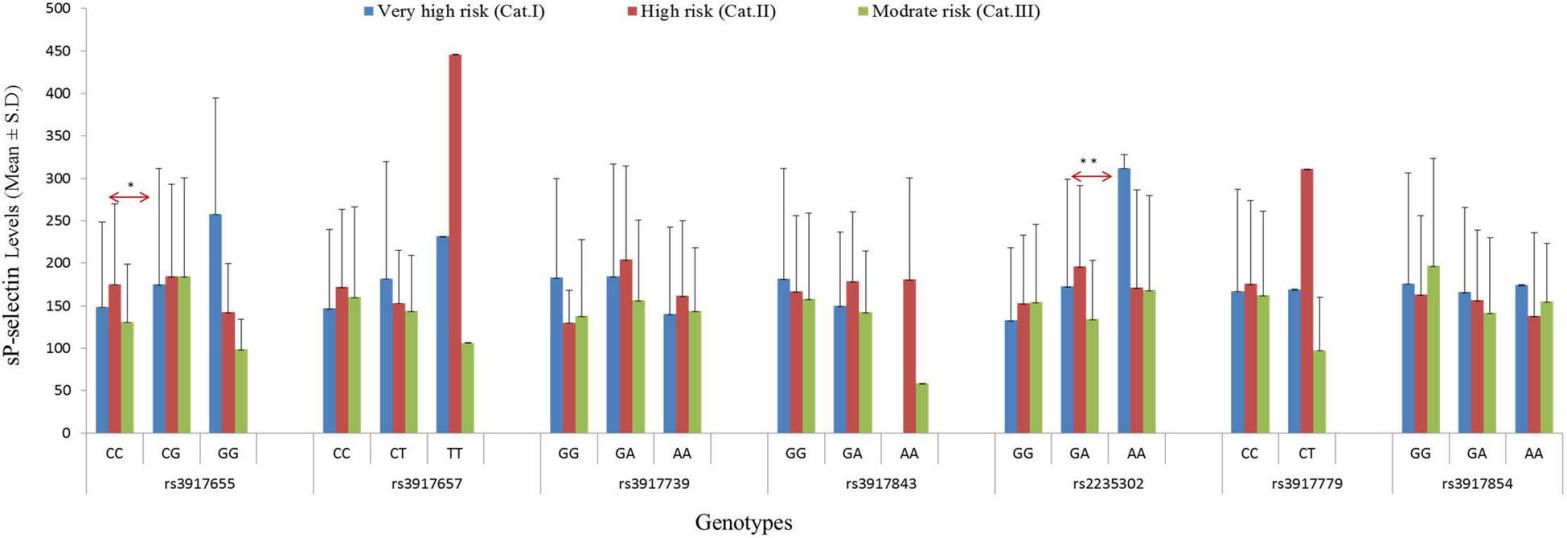
Comparison of sP-selectin levels between vascular risk categories stratified according to different genotypes. Red arrow represents the comparison between the high risk and moderate risk categories; * represents significance at 0.05 level; **represents significance at 0.01 level

Comparison of sP-selectin levels within vascular risk categories revealed significant difference within moderate risk category for rs3917655 variant (*p* < 0.05) (Fig. 3**)**. Comparison between categories revealed significant difference between homozygous wild genotypes in high risk and moderate risk category for rs3917655 (*p* < 0.05), while same pattern was observed in GA genotype for rs2235302 (*p* < 0.001). Furthermore, no statistically significant difference was found in vascular risk categories for other studied variant (*p* > 0.05).

**Fig. 4:**
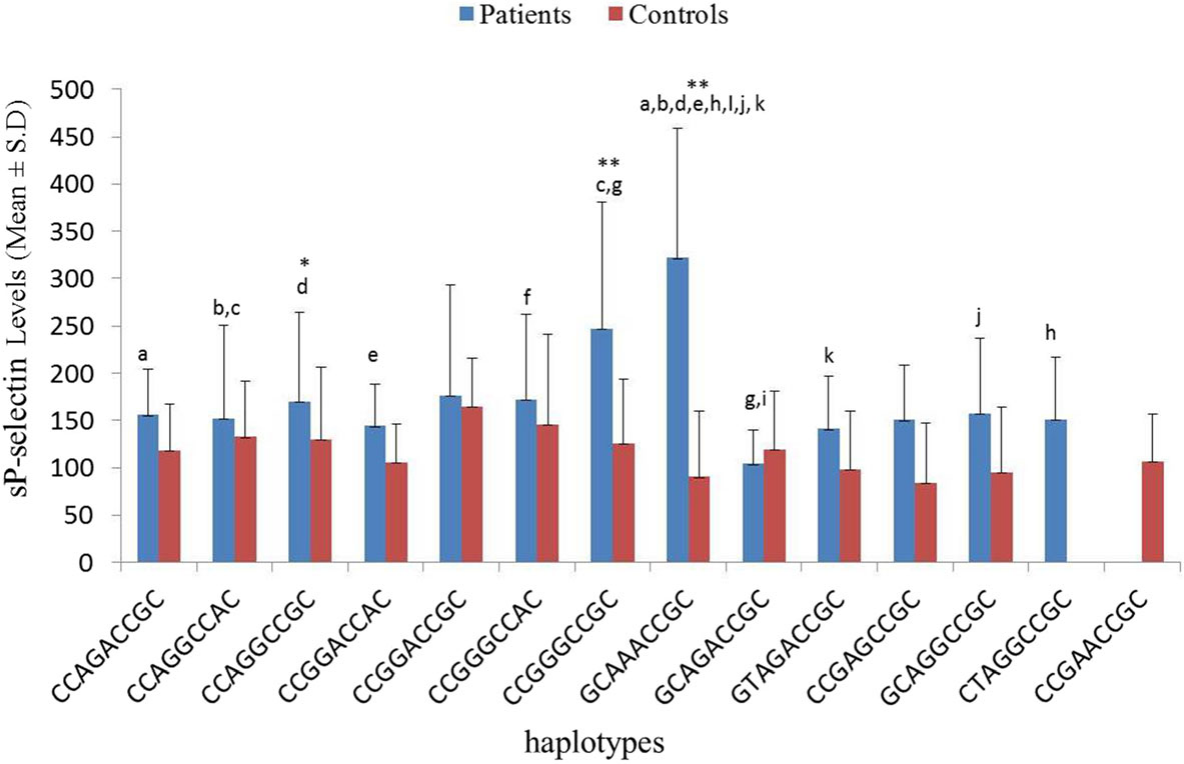
Comparison of sP-selectin levels between T2DM patients and controls segregated according to haplotypes. ^a^*p* = 0.010, ^b^*p* = 0.004; ^c,j^*p* = 0.026, ^d^*p* = 0.017, ^e^*p* = 0.023, ^f^*p* = 0.043, ^g^*p* = 0.032, ^h^*p* = 0.006, ^i^*p* = 0.003, ^k^*p* = 0.007; * represents significance at 0.05 level between the groups; **represents significance at 0.01 level between the groups

sP-selectin levels were also segregated according to haplotypes. Only haplotypes with number of participants more than or equal to five were involved in the present analyses. The criterion of *n* ≥ 5 participants was fulfilled by 12 haplotypes in patients and 11 haplotypes in control with 10 common haplotypes (Fig. 4). Significant difference was observed in sP-selectin levels only within the patient group (*p* > 0.001). Patients with haplotype GCAAACCGC were obserevd to have significantly higher sP-selectin levels than patients with haplotype CCAGACCGC, CCAGGCCAC, CCAGGCCGC, CCGGACCAC, CTAGGCCGC, GCAGACCGC, GCAGGCCGC and GTAGACCGC (*p* < 0.05; 0.01; < 0.05; < 0.05; < 0.01; < 0.01; < 0.01; < 0.05; < 0.01, respectively). In addition, patients with CCGGGCCGC haplotype were found to have significantly raised levels of sP-selectin as compared to patients with haplotype CCAGGCCAC and GCAGACCGC (*p* < 0.05 each). When sP-selectin levels were compared between patients and controls, patients with haplotype GCAAACCGC, CCAGGCCGC and CCGGGCCGC were observed with significantly high sP-selectin levels as compared to controls with respective haplotypes (*p* < 0.01; < 0.05; < 0.01, respectively).

**Fig. 5:**
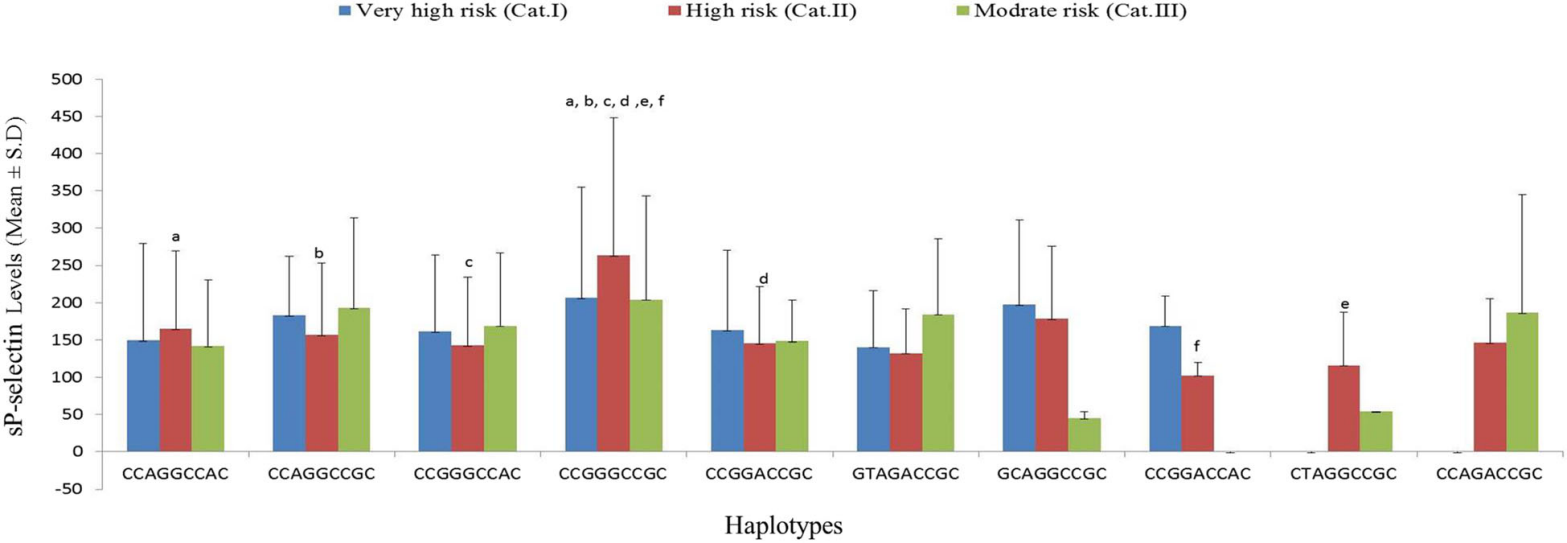
Comparison of sP-selectin levels between vascular risk categories segregated according to non-coding haplotypes. ^a,d^*p* = 0.010, ^b^*p* = 0.003, ^c^*p* = 0.002, ^e^*p* = 0.004, ^f^*p* = 0.005

Segregation of sP-selectin levels according to haplotypes in various vascular risk categories is shown in Fig. 5. A total of 4 haplotypes in very high-risk category and 6 haplotypes each in both high risk and moderate risk category were fulfilled the criterion of participants more than and equal to 5. sP-selectin levels were significantly different only within high risk category, where patients with CCGGGCCGC haplotypes were having significantly elevated sP-selectin levels in comparison to patients with CCAGGCCAC, CCAGGCCGC, CCGGACCGC, CCGGACCAC and CTAGGCCGC haplotypes (*p* < 0.05; < 0.01; < 0.01; < 0.05; < 0.01; < 0.01, respectively). However, no significant difference in sP-selectin levels was found for any of the haplotype when compared between the categories (*p* > 0.05).

## Discussion

T2DM, also known as non–insulin-dependent diabetes or adult-onset diabetes, is accounting for 90–95% of total DM cases worldwide and is the most prevalent form of DM. Adverse effects of chronic hyperglycemia in T2DM are generally divided into microvascular and macrovascular complications. The micro-vascular complications comprised of diabetic retinopathy, neuropathy and nephropathy [58]. The macro-vascular complications are exhibited as accelerated atherosclerosis that results into premature coronary artery disease (CAD), severe peripheral vascular disease and increased risk of cerebrovascular diseases [59–62]. P-selectin, C-type lectin, is known as one of the key markers of platelet activation and endothelial dysfunction. Because of the involvement of initial steps of leukocyte recruitment and thrombus formation, P-selectin has been suggested to play an important role in progression of atherothrombosis, thereby increasing risk of atherosclerotic vascular complications [63, 64]. SELP variants have been suggested as modulators in various inflammatory and atherothrombotic diseases [26, 34–36, 38–41]. Moreover, various *SELP* variants have been reported to influence the levels of soluble P-selectin in different atherosclerotic vascular complications [26, 36, 43–48]. Since the previous studies were mostly focused on missense mutations, the present study employed case-control setup to evaluate the role of nine putative functional non-coding variants of *SELP* in modulation of sP-selectin levels and vascular risk in T2DM. As per literature survey, this is the first research report on study of non-coding SNPs of *SELP* in relation to sP-selectin levels as well as arterial stiffness in T2DM patients in any Asian population.

The clinical relevance of three SNP variants i.e. rs3917655, rs3917853 and rs3917854 has been assessed for first time in the present study. Out of these, only rs3917854 has shown significant association with T2DM as well as vascular risk. Furthermore, both T and C allele carriers were observed to have equal odds of T2DM. Out of the other variants, only three variants i.e. rs3917657, rs2235302, rs3917779 were found to be associated with different disease conditions. In a Genome- wide linkage study including UK and USA populations, a stronger association of rs3917657 was observed with SLE [49]. Another important non-coding SNP rs2235302 is located between consensus repeat (CR) 3 and CR4. In the present study, variant allele of rs2235302 was found to be protective. Furthermore, carriers of G allele have been shown to be associated with equal odds of T2DM as carriers with A allele. However, this variant was shown to be associated with increased thickness of carotid intima media in a previous study [50]. The 3rd important variant i.e. rs3917779 is located in the intron 10 at binding site of transcriptional repressor CTCF (CCCTC-binding factor), known to be involved in various regulatory activities [65, 66]. It was associated with the development of proliferative diabetic retinopathy in Iranian population [51]. The study suggested that TT genotype of rs3917779 may abolish CCCTC- binding factor binding site, thus affect the transcription [51]. In the present genetic association study, no variant genotype (TT) was observed in any of the studied group. Furthermore, no statistically significant association was observed with T2DM and vascular risk. In addition, the patterns of pairwise LD displayed by *SELP* polymorphisms suggested the existence of highly conserved haplotypes.

After performing genotypic analyses of all the studied SNPs, haplotypes were constructed. The haplotype-based approaches have several advantages over the traditional genotype-based strategies [67]. Haplotypes may have specific significance with respect to functionability or as markers for unidentified functional variations. The haplotype-based approach may provide a better tool to distinguish haplotype from a single variant and to determine whether the influence of the variant dependent upon the haplotypic background by which it is carried or not. Moreover, the candidate genes are further translated into polypeptides, which may structurally and functionally dependent on the presence of various amino acids. Thus, for better depiction of role of a candidate gene, the full exploitation of haplotypic information is very important [68, 69]. Only GCAGGCCGC haplotype was observed at significantly high frequency in T2DM patients as compared to controls, conferring 4.1 -fold risk of disease development. In this haplotype, seven out of nine alleles were wild alleles except for rs3917655 (G) and rs3917739 (C). Both of these variants were observed to be in LD with rs3917657, associated with 2 -fold risk of disease development. Evolutionary conservation of rs3917655G and rs3917739C alleles (and its adjoining sequence) provided tentative evidence for their functionality. There are only two reports showing haplotype distribution of *SELP* variants in T2DM patients [26, 70].

Furthermore, no statistically significant difference was obtained in frequencies of non-coding haplotype between the vascular risk categories. Previous studies suggested that various haplotypes of *SELP* polymorphisms may be established as the predictive marker in the etiology of various diseases including MI, CHD, SLE, venous thromboembolism, recurrent spontaneous abortions [35, 39, 40, 49, 67, 71]. As per literature survey, this is the first comprehensive study involving the genotypic and haplotypic analyses of putative functional non-coding variants of *SELP* in T2DM as well as vascular risk categories.

A genotypic-phenotypic correlation analyses was also executed for *SELP* variants and haplotypes in the studied groups. Association of *SELP* variants and haplotypes has earlier been assessed with sP-selectin levels in different disease conditions [26, 36, 44–48]. Overall sP-selectin levels were higher in T2DM patients when segregated according to genotypes as well as haplotypes. There are only two reports showing significant association of one non-coding variant (rs2235302) with higher sP-selectin levels [48, 50]. Significant genotype-phenotype correlations were observed for rs3917655 as well as rs3917739 variant within patients and for rs3917854 within controls.

Furthermore, sP-selectin levels were also segregated according to *SELP* haplotypes. Patients with GCAAACCGC haplotype, containing variant allele of rs3917655, rs3917739, rs3917843 and rs2235302, were observed with significantly increased levels of sP-selectin than patients with haplotype CCAGACCGC, CCAGGCCAC, CCAGGCCGC, CCGGACCAC, CTAGGCCGC, GCAGACCGC, GCAGGCCGC, GTAGACCGC and controls with the GCAAACCGC haplotype. When studied individually, all these four SNPs rs3917655, rs3917739, rs3917843 and rs2235302 were also accounted for high sP-selectin levels in patients than controls. Variant allele of rs3917843, associated with GCAAACCGC haplotype, may account for significantly high level of sP-selectin, because of its absence in other haplotypes. Furthermore, haplotype CCGGGCCGC containing all the wild alleles was also observed with significantly high sP-selectin levels in patients as compared to patients with haplotype CCAGGCCAC and GCAGACCGC and controls with alike haplotypes. This is the first report showing the genotypic and haplotypic association of non-coding *SELP* polymorphisms in T2DM as well as vascular risk categories.

A question however arises as to what the possible explanation for these SELP variants in risk is as well as protection towards disease development. In silico analyses of the majority of the SNPs investigated in the present study showed their regulatory effect by altering the transcription factor (TF) binding site activity [53]. Furthermore, the SNPs localized in close proximity to promoter can cause significant alterations in TFs binding, downregulating *SELP* transcription and thus affecting intitial steps of adhesion cascade. In addition, glucose and lipid lowering therapies have been indicated as potential factors modulating CVD risk in T2DM [72, 73]. Further studies are warranted to validate these assumptions.

However, there are some limitations in the present study. Although, the present sample size had a sufficient statistical power i.e. 94% for performing the genetic analyses, the study was insufficiently powered for the vascular risk categories. Furthermore, baPWV, being an expensive method, could not to be performed in controls. In addition to address these limitations, further studies can be planned to assess contribution of glucose and lipid lowering therapies on CVD risk in T2DM.

## Conclusion

The present study indicated significant modulation of sP-selectin levels, vascular risk and T2DM susceptibility, associated with non-coding *SELP* variants. The findings of this study may provide promising basis for understanding genotype-phenotype correlation in the pathogenesis of complex disease conditions and develop protocols for intervention strategies. In addition, our findings strongly indicate that non-coding polymorphisms of *SEL*P may serve as novel molecular biomarkers for early prediction as well as screening of vascular risk and even as potential therapeutic targets. The outcomes of the present study provide a rationale for extensive screening of SELP variants in the diverse populations.

## Supplementary information

**Additional file 1: Figure S1.** (a) Representative agarose gel showing PCR product of size 405 bp and restriction digestion products obtained for rs3917655. **Figure S2.** (a) Representative agarose gel showing PCR product of size 516 bp and restriction digestion products obtained for rs3917657. **Figure S4.** (a) Representative agarose gel showing PCR product of size 578 bp and restriction digestion products obtained for rs3917843. **Figure S5.** (a) Representative agarose gel showing PCR product of size 511 bp and restriction digestion products obtained for rs2235302. **Figure S6.** (a) Representative agarose gel showing ARMS-PCR products of size 441, 254 and 243 bp for rs3917779. **Figure S7.** (a) Representative agarose gel showing PCR product of size 620 bp obtained after amplification of gene region showing rs3917853, rs3917854 and rs3917855; (b) Electropherograms of representative samples of rs3917853 confirming homozygous wild genotype; (c) Electropherograms of representative samples of rs3917855 confirming homozygous wild genotype; (d)Electropherograms of representative samples of rs3917854 confirming homozygous wild genotype; (e) homozygous variant genotype and (f) heterozygous genotype.

**Additional file 2: Table S1.** Details of various components used in PCR-RFLP of rs3917655, rs3917657, rs3917739, rs3917843 and rs2235302. **Table S2.** PCR components and their concentrations used for rs3917779.

## Data Availability

The datasets used and/or analyzed during the current study are available from the corresponding author on reasonable request.

## Abbreviations

ARMS: Amplification-refractory mutation system
baPWV: Brachial-ankle pulse wave velocity
CAD: Coronary artery disease
CHD: Coronary heart disease
CR: Consensus repeat
DNA: Deoxyribonucleic acid
EDTA: Ethylenediaminetetraacetic acid
EtBr: Ethidium bromide
ELISA: Enzyme-linked immunosorbent assay
HbA1c: lycated hemoglobin
ICMR: Indian Council of Medical Research guidelines
IDF: International Diabetes Federation
LD: Linkage disequilibrium
MI: Myocardial infarction
PAD: Peripheral artery disease
PB: Punjab
PCR: Polymerase chain reaction
RFLP: Restriction fragment length polymorphism
SNPs: Single-nucleotide polymorphisms
sP-selectin: soluble P-selectin
SPSS: Statistical package for Social science
T2DM: Type 2 diabetes mellitus
