# Supplementary material for: Putative functional non-coding polymorphisms in SELP significantly modulate sP-selectin levels, arterial stiffness and type 2 diabetes mellitus susceptibility
Source: BMC Endocr Disord. 2020 May 19;20:70. doi: 10.1186/s12902-020-00548-x (PMC7236446; doi:10.1186/s12902-020-00548-x)
Supplement: Supplementary file 1 — Additional file 1: Figure S1. (a) Representative agarose gel showing PCR product of size 405 bp and restriction digestion products obtained for rs3917655. Figure S2. (a) Representative agarose gel showing PCR product of size 516 bp and restriction digestion products obtained for rs3917657. Figure S4. (a) Representative agarose gel showing PCR product of size 578 bp and restriction digestion products obtained for rs3917843. Figure S5. (a) Representative agarose gel showing PCR product of size 511 bp and restriction digestion products obtained for rs2235302. Figure S6. (a) Representative agarose gel showing ARMS-PCR products of size 441, 254 and 243 bp for rs3917779. Figure S7. (a) Representative agarose gel showing PCR product of size 620 bp obtained after amplification of gene region showing rs3917853, rs3917854 and rs3917855; (b) Electropherograms of representative samples of rs3917853 confirming homozygous wild genotype; (c) Electropherograms of representative samples of rs3917855 confirming homozygous wild genotype; (d)Electropherograms of representative samples of rs3917854 confirming homozygous wild genotype; (e) homozygous variant genotype and (f) heterozygous genotype. [file 12902_2020_548_MOESM1_ESM.docx]

**
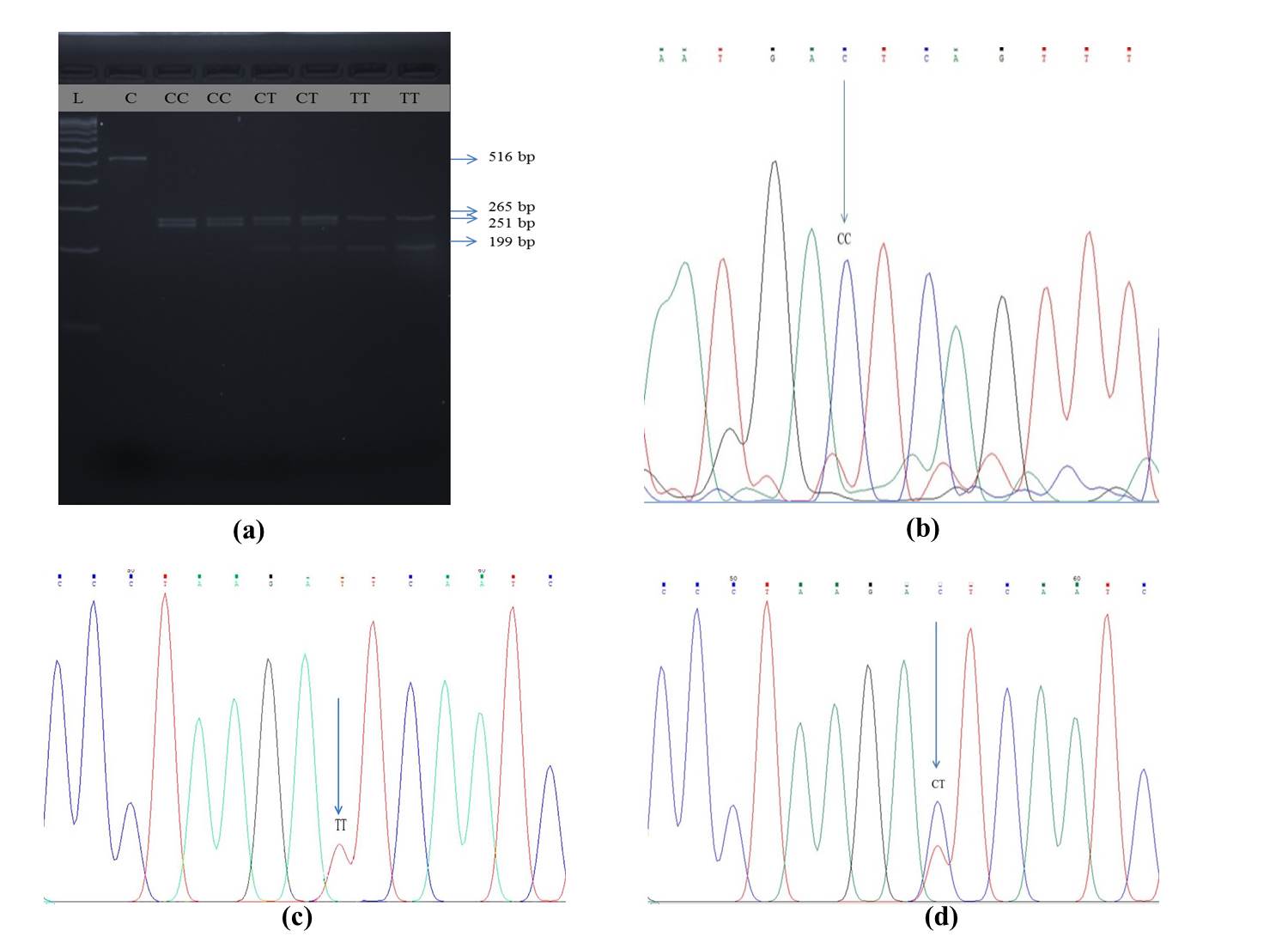
**

**Supplementary Figure 1:** (a) Representative agarose gel showing PCR product of size 405 bp and restriction digestion products obtained for rs3917655. L represents 100bp ladder, C represents negative control and products obtained were 249, 156 bp for CC genotype, 405 bp for GG genotype and 405, 249 bp and 156 bp for CG genotype; (b) Electropherograms of representative samples of rs317655 confirming homozygous wild genotype (c) homozygous variant genotype and (d) heterozygous genotype.

**
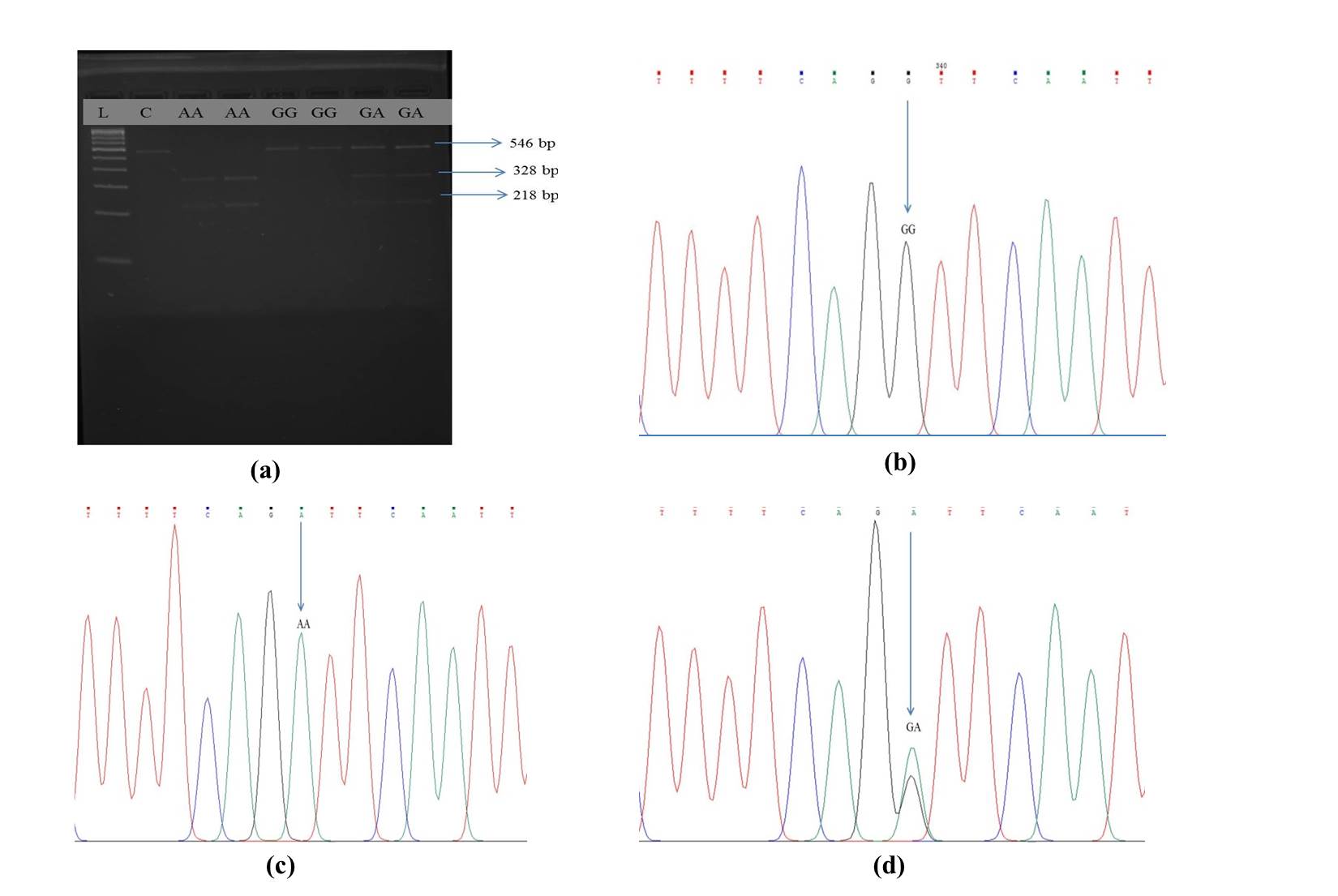
**

**Supplementary Figure 2:** (a) Representative agarose gel showing PCR product of size 516 bp and restriction digestion products obtained for rs3917657. L represents 100bp ladder, C represents negative control and products obtained were 265, 251 bp for CC genotype; 265, 199, 52 bp for TT genotype and 405, 265, 199, 52 bp for CT genotype; (b) Electropherograms of representative samples of rs3917657 confirming homozygous wild genotype; (c) homozygous variant genotype and (d) heterozygous genotype.


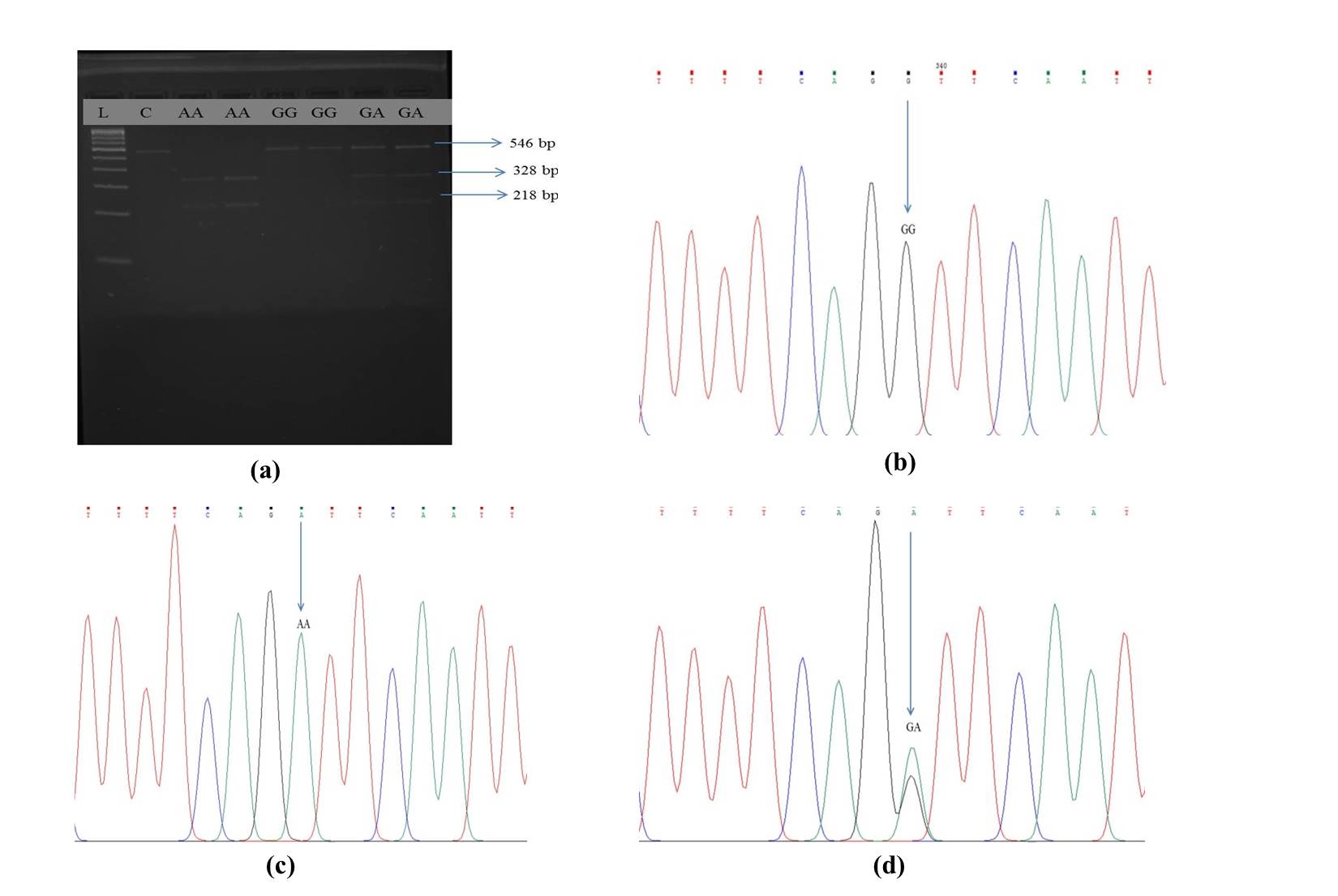


**Supplementary Figure 3:** (a) Representative agarose gel showing PCR product of size 546 bp and restriction digestion products obtained for rs3917739. L represents 100bp ladder, C represents negative control and products obtained were 546 bp for GG genotype; 328, 218 bp for AA genotype and 546, 328, 218 bp for GA genotype; (b) Electropherograms of representative samples of rs3917739 confirming homozygous wild genotype; (c) homozygous variant genotype and (d) heterozygous genotype.


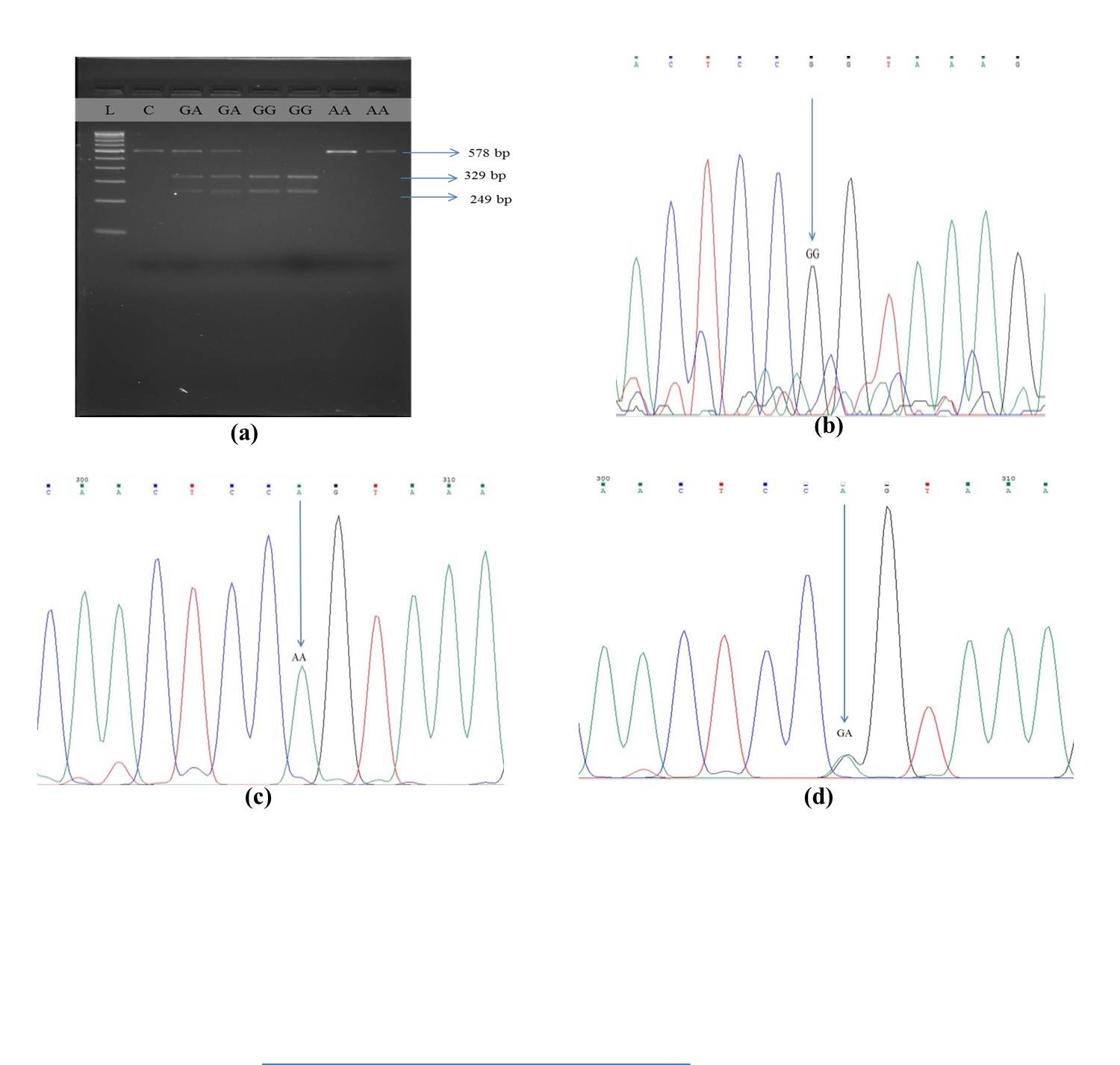


**Supplementary figure 4:** (a) Representative agarose gel showing PCR product of size 578 bp and restriction digestion products obtained for rs3917843. L represents 100bp ladder, C represents negative control and products obtained were 329, 249 bp for GG genotype; 578 bp for AA genotype and 578, 329, 249 bp for GA genotype; (b) Electropherograms of representative samples of rs3917843 confirming homozygous wild genotype (c) homozygous variant genotype and (d) heterozygous genotype.


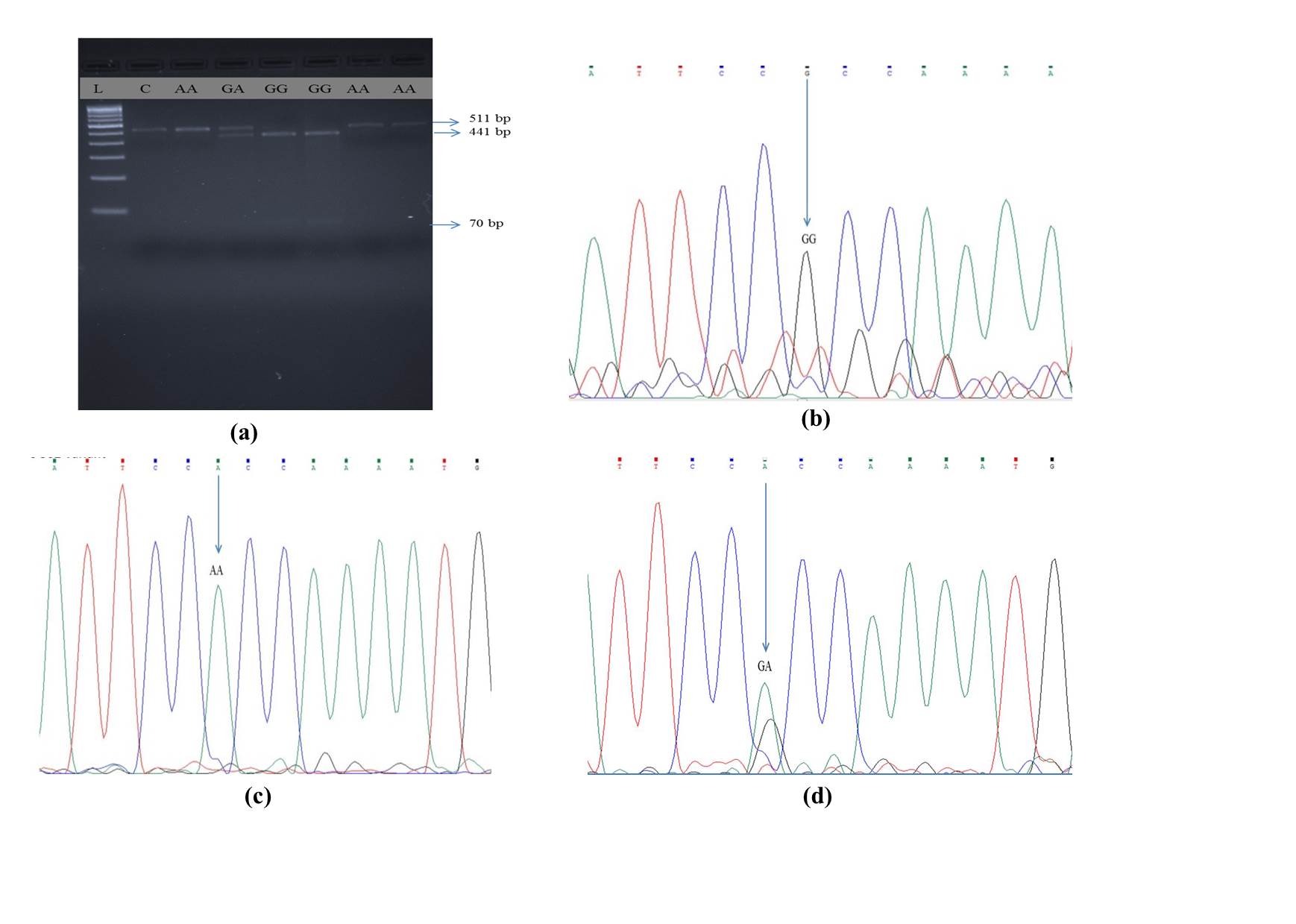
**Supplementary figure 5:** (a) Representative agarose gel showing PCR product of size 511 bp and restriction digestion products obtained for rs2235302. L represents 100bp ladder, C represents negative control and products obtained were 441, 70 bp for GG genotype; 511 bp for AA genotype and 511, 441, 70 bp for GA genotype; (b) Electropherograms of representative samples of rs2235302 confirming homozygous wild genotype; (c) homozygous variant genotype and (d) heterozygous genotype.


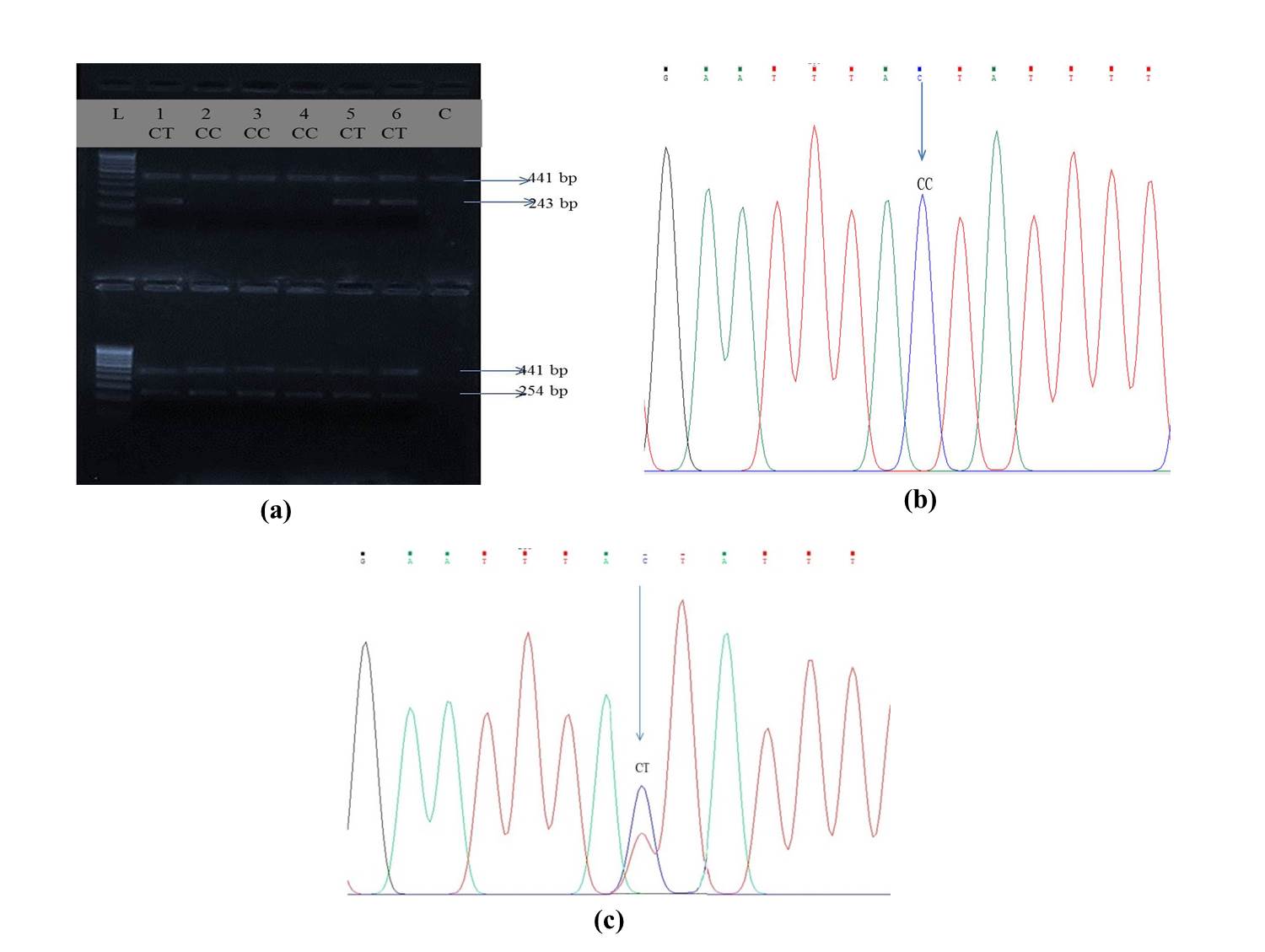


**Supplementary figure 6:** (a) Representative agarose gel showing ARMS-PCR products of size 441, 254 and 243 bp for rs3917779. L represents 100bp ladder, C represents negative control and products obtained were 441, 254 and 243bp for CT genotype; 441 and 254 bp for CC genotype; (b) Electropherograms of representative samples of rs3917779 confirming homozygous wild genotype and (c) heterozygous genotype.


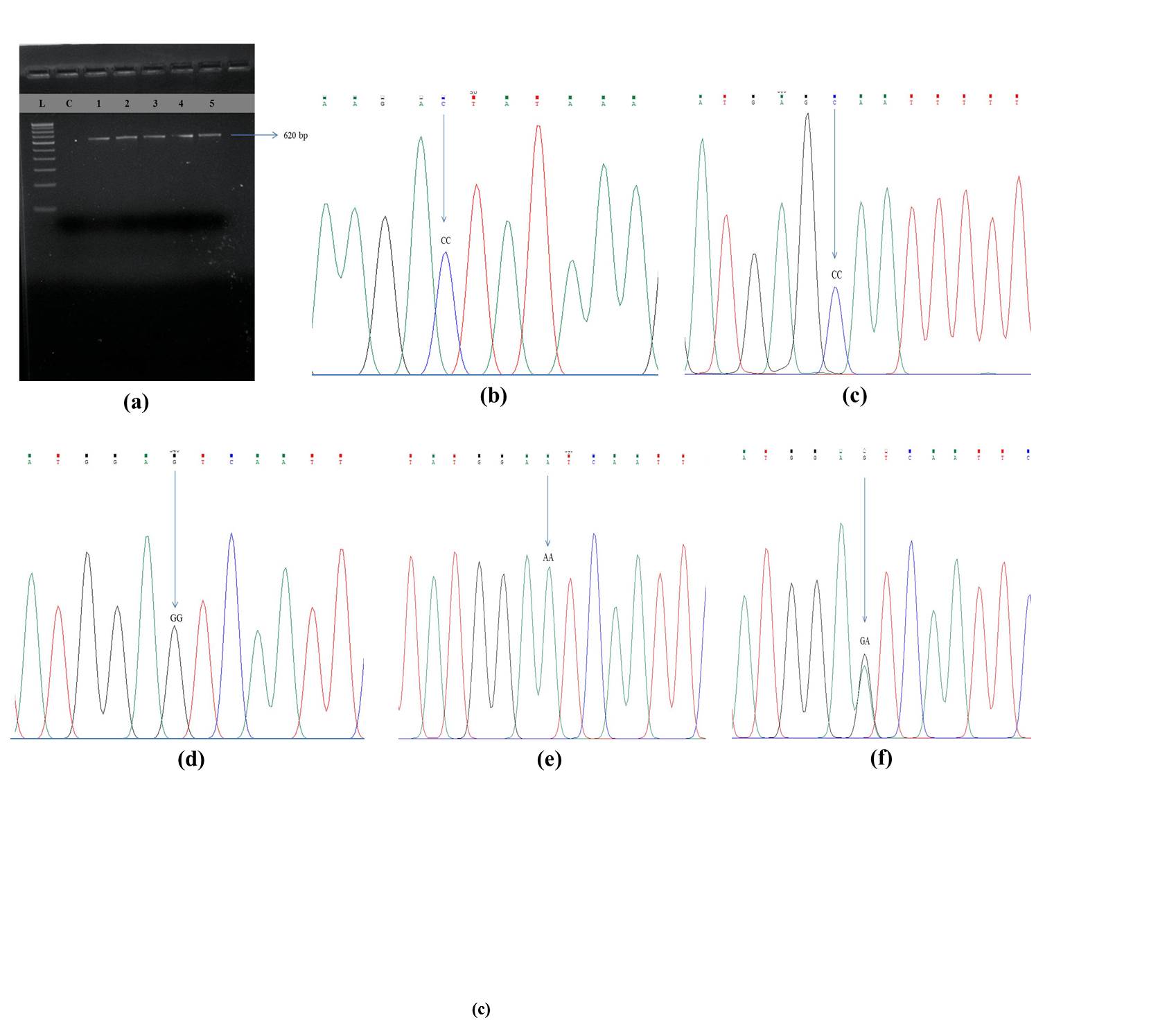


**Supplementary figure 7:** (a) Representative agarose gel showing PCR product of size 620 bp obtained after amplification of gene region showing rs3917853, rs3917854 and rs3917855; (b) Electropherograms of representative samples of rs3917853 confirming homozygous wild genotype; (c) Electropherograms of representative samples of rs3917855 confirming homozygous wild genotype ;(d)Electropherograms of representative samples of rs3917854 confirming homozygous wild genotype; (e) homozygous variant genotype and (f) heterozygous genotype.
