# Supplementary material for: Putative functional non-coding polymorphisms in SELP significantly modulate sP-selectin levels, arterial stiffness and type 2 diabetes mellitus susceptibility
Source: BMC Endocr Disord. 2020 May 19;20:70. doi: 10.1186/s12902-020-00548-x (PMC7236446; doi:10.1186/s12902-020-00548-x)
Supplement: Supplementary file 2 — Additional file 2: Table S1. Details of various components used in PCR-RFLP of rs3917655, rs3917657, rs3917739, rs3917843 and rs2235302. Table S2. PCR components and their concentrations used for rs3917779. [file 12902_2020_548_MOESM2_ESM.docx]

**Supplementary table 1: Details of various components used in PCR-RFLP of rs3917655, rs3917657, rs3917739, rs3917843 and rs2235302**

| **SNP** | **Concentration of stock solution** | | **Concentration of working solution** | | **Volume of working solution used to make final volume of 5 µl (µl)** | | |
| --- | --- | --- | --- | --- | --- | --- | --- |
|  | **Restriction enzyme (concentraction)** | **NEBuffer**  **Or**  **CutSmart® Buffer** | **Restriction enzyme (concentraction)** | **NEBuffer**  **or**  **CutSmart® Buffer** | **Restriction enzyme** | **NEBuffer or CutSmart® Buffer** | **Deionized water** |
| rs3917655 | *PvuII* | 10 X | 1U | 1 X | 0.1 | 0.5 | 4.4 |
|  | (10000 U/ml) |  |  |  |  |  |  |
| rs3917657 | *TfiI* | 10 X | 1U | 1 X | 0.1 | 0.5 | 4.4 |
|  | (10000 U/ml) |  |  |  |  |  |  |
| rs3917739 | *TfiI* | 10 X | 1U | 1 X | 0.1 | 0.5 | 4.4 |
|  | (10000 U/ml) |  |  |  |  |  |  |
| rs3917843 | *BsaWI* | 10 X | 1U | 1 X | 0.1 | 0.5 | 4.4 |
|  | (10000 U/ml) |  |  |  |  |  |  |
| rs2235302 | *EciI* | 10 X | 1U | 1 X | 0.5 | 0.5 | 4.0 |
|  | 2000 U/ml |  |  |  |  |  |  |

**Supplementary table 2: PCR components and their concentrations used for rs3917779**

| **Components** | **Concentration of Stock solution** | **Concentration of working solution** | **Volume of working solution**  **(20 µl) (C allele)** | **Volume of working solution**  **( 20 µl) (T allele)** |
| --- | --- | --- | --- | --- |
| *Taq* Buffer | 10 X | 1X | 2.0 | 2.0 |
| dNTP mix | 2.5 mM | 0.2mM | 1.6 | 1.6 |
| Primer mix 1  Primer mix 2 | 10 µM  10 µM | 3 µM  3 µM | 0.6  0.6 | 0.6  0.6 |
| *Taq* DNA polymerase | 3 U | 0.3 U | 0.1 | 0.1 |
| Template DNA | 50 ng/ µl | -- | 1 | 1 |
| Deionized water | -- | -- | 14.1 | 14.1 |

Primer mix 1: forward outer + reverse outer; Primer mix 2: forward outer +reverse inner (specific for C allele), and Forward inner (specific for T allele) + reverse outer
